# Mechanical Performance and Marginal Integrity Evaluation of Contemporary Universal Resin Composites for Dental Restorations: An In-Vitro Study

**DOI:** 10.7759/cureus.104424

**Published:** 2026-02-27

**Authors:** Shahzad Ali Shah

**Affiliations:** 1 Conservative Dental Sciences Department, College of Dentistry, Qassim University, Buryadah, SAU

**Keywords:** composite resin, dental material, invitro studies, marginal adaptation, mechanical stress

## Abstract

Background: Universal resin composites are widely used for dental restorations, but differences in resin chemistry, filler composition, and polymerization can affect performance and marginal integrity.

Objective: To evaluate four modern universal resin composites in terms of their mechanical characteristics and marginal integrity.

Materials and methods: Ninety-six specimens were prepared into four groups (UC-A, UC-B, UC-C, UC-D; n = 24 each). Bar and disc shaped specimens were tested for flexural strength, Vickers’ Microhardness, and two-body wear, respectively. Extracted human molars (n = 48) with Class II cavities were restored with composites, marginal integrity using dye penetration (0-3 scoring), and gap width measurement after thermocycling (5,000 cycles). Data were analyzed with one-way ANOVA/Tukey or Kruskal-Wallis/Dunn tests (α = 0.05).

Results: UC-A exhibited the highest flexural strength (125.4 ± 7.8 MPa) and modulus (6.2 ± 0.4 GPa), significantly greater than UC-C (112.7 ± 5.9 MPa; 5.5 ± 0.4 GPa, p < 0.05). Microhardness ranged from 75.4 ± 2.2 VHN (UC-C) to 81.5 ± 2.6 VHN (UC-A, p < 0.01). Wear volume loss was lowest for UC-A (0.087 ± 0.012 mm³) and highest for UC-C (0.108 ± 0.013 mm³, p = 0.04). Marginal integrity was highest for UC-A, with 50% leakage-free restorations and gap width (45.3 ± 6.2 µm), while UC-C had more leakage (Score 3 in 8.3% of cases) and wider gaps (56.9 ± 7.1 µm, p = 0.01). UC-B and UC-D showed intermediate results.

Conclusion: UC-A demonstrated superior mechanical performance and marginal sealing, whereas UC-C performed least favorably, highlighting the influence of resin formulation.

## Introduction

Resin composites are the most widely used direct restorative materials in modern dentistry due to their superior aesthetics, adhesive properties, and ability to maintain tooth structure. With improved handling, strength in stress-bearing regions, and shade flexibility, universal composites are designed for both anterior and posterior applications and have simplified restorative treatments [1,2]. Despite these advantages, differences in filler type, particle size distribution, and resin matrix chemistry may affect the clinical performance of commercially available universal composites, particularly with relation to mechanical strength and marginal adaptability [3,4].

Mechanical characteristics such as wear resistance, microhardness, and flexural strength are important indicators of composite restoration lifetime. These elements determine the material's functional life, resistance to masticatory stresses, and resistance to surface deterioration. Although new developments in filler technology, such as nano-hybrid and supra-nano particles, promise to improve these properties, comparative data among universal composites are still limited and sometimes contradictory [5,6].

Equally important is the composite restorations' marginal integrity. Polymerization shrinkage and interfacial stresses can damage the tooth restoration interface, leading to microleakage, postoperative sensitivity, marginal discoloration, and recurrent caries, which is the most common cause of restoration failure [7,8]. Improvements in filler loading and photo initiator technology are meant to reduce polymerization shrinkage stress, even though universal composites behave quite differently along the borders of Class II cavities.

Although a number of studies have independently evaluated mechanical behavior or marginal adaptability, few have directly investigated the number of universal composites under controlled laboratory conditions [9]. Therefore, in order to give clinicians evidence-based recommendations for selecting the most dependable universal composite for long-term success, a thorough in-vitro evaluation of both mechanical and marginal integrity characteristics is required. The aim of the current investigation was to compare the mechanical properties and marginal integrity of four widely available universal composites. This study aimed to produce reliable data that illustrate variations in material performance and aid in restorative decision-making by mimicking standardized clinical circumstances in an in vitro environment.

## Materials and methods

This in-vitro comparative experimental study evaluated the mechanical properties and marginal integrity of four contemporary universal resin composites. The study was conducted in the Conservative Dental Sciences Department of Qassim University under controlled conditions. Four universal resin composites (shade A2) that are sold commercially were chosen: Group A, Universal composite A (Nano-hybrid) (UC-A), Group B, Universal composite B (Nano-filled) (UC-B), Group C, Universal composite C (Supra-Nano-filled) (UC-C), and Group D, Universal composite D (Bulk-fill type) (UC-D).

For each group, a single light-curing unit (light-emitting diode (LED); ≥1000 mW/cm^2^, radiometer-calibrated) and a standardized adhesive system were used to minimize variability. The four groups received equal distributions of the 96 prepared specimens (n = 24/each group). Mechanical testing of specimens in the shapes of discs and bars: n = 12 per group. n = 12 specimens of marginal integrity (restored teeth removed) in each group.

**Figure 1:**
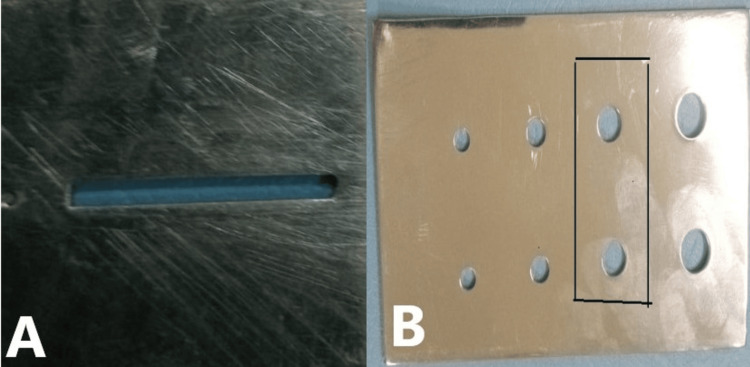
Composite sample preparation A. Rectangular bar (ISO 4049) and B. Disc shape SS mold (outlined).

Flexural strength and microhardness: Rectangular bars (25 × 2 mm) were fabricated using stainless steel molds in accordance with ISO 4049 standards (Figure 1A). Composites were inserted incrementally, covered with Mylar strips and glass slides, and light-cured for 20 seconds per increment. Disc-shaped specimens (6 mm diameter, 2 mm thickness) were prepared. After curing, the discs were polished with 600-grit silicon carbide paper to standardize the surface (Figure 1B). After preparation, all specimens were stored in distilled water at 37 °C for 24 hours before testing.

Marginal integrity specimens

**Figure 2:**
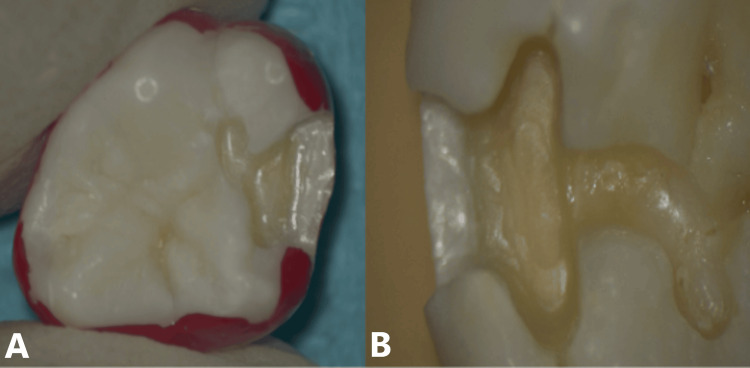
Study samples A. Class II cavity with occlusal dovetail in maxillary molar and B. Mandibular molar.

Forty-eight (48) human molars/premolars extracted free from caries, cracks, or restorations were collected, cleaned, and stored in 0.1% thymol solution at 4 °C. Standardized Class II cavities (4 mm Bucco-lingual width, 4 mm Occluso-gingival height, 2 mm axial depth) were prepared on the proximal surfaces with occlusal dovetails were prepared using a high-speed hand piece with #245 tungsten carbide bur, under water coolant (Figure 2).

**Figure 3:**
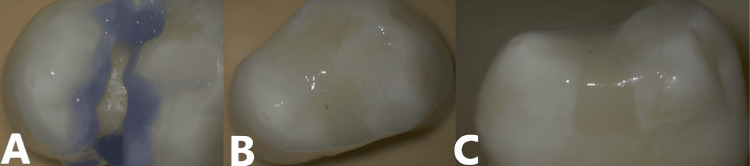
Sample preparation procedure A. Selective etching with 37% phosphoric acid, B. Composite restoration following manufacturer instructions (occlusal view), and C. Proximal view.

Each cavity was etched (37% phosphoric acid, 15 sec), rinsed, and dried. The adhesive was applied according to the manufacturer’s instructions, followed by incremental composite placement and light curing (Figure 3).

**Figure 4:**
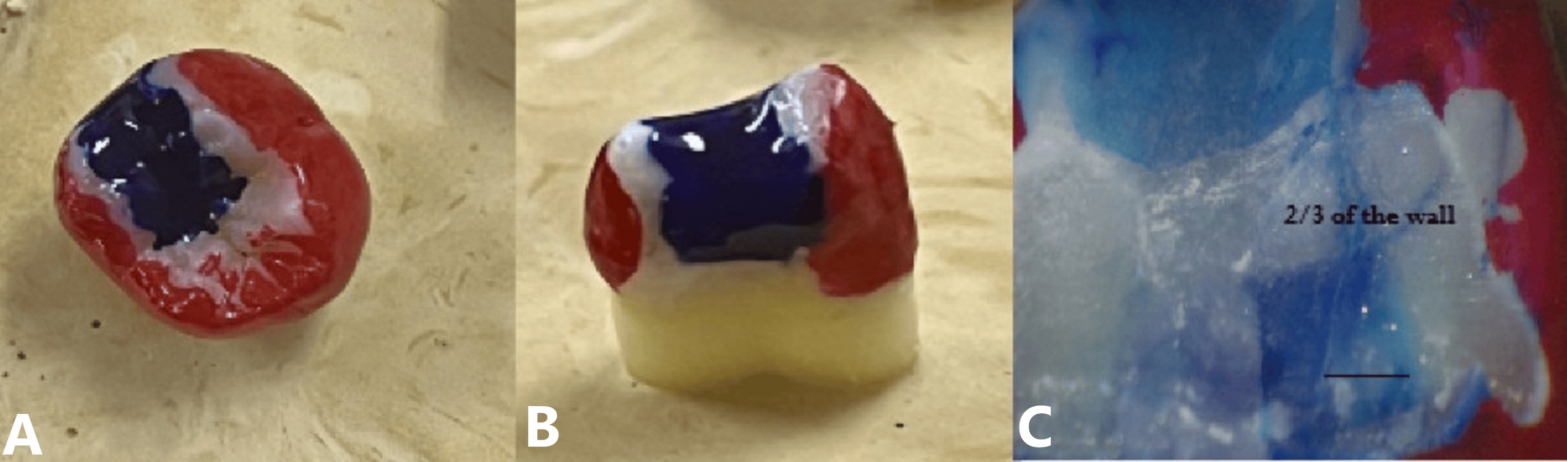
Dye penetration test for marginal integrity A. Occlusal view B. Proximal view C. 20X magnified view of dye penetration showing more than 2/3rd of dentin.

Aging and dye penetration: After 24 h of water storage, specimens underwent thermocycling (5,000 cycles, 5-55 °C, 30 s dwell time). The teeth were then coated with nail varnish, leaving 1 mm around the restoration margin, immersed in 0.5% basic fuchsin dye for 24 h (Figure 4), sectioned Bucco-lingually, and examined under a 20X digital operating microscope (Zumax Medical Co., Ltd., Suzhou, China).

Testing procedures

The samples were tested using the following test methods: (a) Flexural strength and modulus: the samples were tested in a Universal Testing Machine (crosshead speed 1 mm/min), and flexural strength (MPa) and modulus (GPa) were calculated; (b) Vickers microhardness: three indentations per disc were made using a microhardness tester (load 200 g, dwell time 15 sec), and mean values were recorded in Vickers Hardness Number (VHN); (c) Marginal integrity: dye penetration was scored using a 0-3 ordinal scale (0 = no penetration; 1 = penetration up to 1/3 of wall; 2 = penetration up to 2/3 of wall; 3 = penetration to axial wall). Gap width (µm) was also measured at three points per section under 20× magnification using a digital operating microscope (Zumax Medical Co. Ltd., Suzhou, China).

Data was analyzed using the statistical software SPSS, version 26 (IBM Corp., Armonk, NY). Normality was assessed using the Shapiro-Wilk test. One-way ANOVA with Tukey’s post hoc test was applied for continuous variables (flexural strength, modulus, Microhardness, and gap width). The Kruskal-Wallis test followed by Dunn’s post hoc test was used for ordinal dye penetration scores. Statistical significance was set at p < 0.05.

## Results

The results of this study indicate that UC-A (Nano-hybrid) consistently outperformed the other tested materials in both mechanical robustness and marginal sealability.

Mechanical performance

The evaluation of flexural strength revealed a significant hierarchy, with UC-A achieving the highest mean value of 125.4 ± 7.8 MPa, which was significantly superior to UC-C at 112.7 ± 5.9 MPa (p < 0.05). This trend was mirrored in the flexural modulus (stiffness) data, where UC-A recorded 6.2 ± 0.4 GPa compared to the significantly lower 5.5 ± 0.4 GPa found in UC-C. Surface resistance, measured via Vickers microhardness, also peaked with UC-A at 81.5 ± 2.6 VHN, whereas UC-C demonstrated the lowest resistance at 75.4 ± 2.2 VHN (p < 0.01). Furthermore, UC-A exhibited the greatest wear resistance, with a significantly lower volume loss (0.087 mm³) than UC-C (0.108 mm³, p = 0.04) (Table 1).

**Table 1:** Mechanical performance of universal composites (one-way ANOVA with Tukey’s post hoc analysis) Data are presented as mean ± standard deviation (n = 12 per group). Normality was confirmed using the Shapiro–Wilk test. Statistical analysis was performed using one-way ANOVA followed by Tukey’s HSD post hoc test. Flexural strength: F(3,44) = 8.92, p < 0.001, Flexural modulus: F(3,44) = 7.18, p = 0.001, Microhardness: F(3,44) = 12.46, p < 0.001, Wear volume loss: F(3,44) = 3.12, p = 0.034, Post hoc analysis revealed that UC-A showed significantly higher flexural strength, modulus, and microhardness compared to UC-C (p < 0.05).

Composite Group	Flexural Strength (MPa) ± SD	Flexural Modulus (GPa) ± SD	Microhardness (VHN) ± SD	Wear Volume Loss (mm³) ± SD
UC-A	125.4 ± 7.8	6.2 ± 0.4	81.5 ± 2.6	0.087 ± 0.012
UC-B	119.8 ± 6.5	5.9 ± 0.3	78.9 ± 2.4	0.093 ± 0.011
UC-C	112.7 ± 5.9	5.5 ± 0.4	75.4 ± 2.2	0.108 ± 0.013
UC-D	117.6 ± 6.1	5.8 ± 0.3	77.1 ± 2.5	0.097 ± 0.010

Marginal integrity

Marginal Gap Width

The marginal sealability of the restorations, assessed after 5,000 thermocycles, showed that UC-A provided the most stable interface, yielding the smallest mean gap width (45.3 ± 6.2 µm). In contrast, UC-C exhibited significantly wider gaps (56.9 ± 7.1 µm, p = 0.01). Dye penetration scores supported these findings; 50% of UC-A restorations remained entirely leakage-free (Score 0), with no cases of severe penetration. Conversely, UC-C was the most susceptible to failure, with only 33% of specimens remaining leakage-free and a notable instance of dye reaching the axial wall (Score 3 in 8.3% of cases) (Table 2). In summary, across all outcomes, UC-A demonstrated superior structural integrity, while UC-C performed the least favorably, with UC-B and UC-D maintaining intermediate, clinically acceptable results.

**Table 2:** Marginal integrity of tested universal composites (one-way ANOVA followed by Tukey’s post hoc test) Data are presented as mean ± standard deviation (n = 12 per group). Marginal gap width was analyzed using one-way ANOVA with Tukey’s post hoc test: Gap width F(3,44) = 7.40, p = 0.0004. Dye penetration scores (ordinal data) were analyzed using the Kruskal–Wallis test followed by Dunn’s post hoc test: Leakage score H(3) = 9.87, p = 0.020. Groups sharing the same letter are not significantly different (Tukey/Dunn post hoc, p > 0.05).

Composite Group (n=48)	Mean Gap Width (µm) ± SD	Leakage Score (0–3)	%Leakage-Free Restorations (Score 0)	Significance
UC-A (n=12)	45.3 ± 6.2	0.6 ± 0.4	50%	a
UC-B (n=12)	49.2 ± 5.8	0.9 ± 0.5	41.7%	a, b
UC-C (n=12)	56.9 ± 7.1*	1.3 ± 0.6*	33.3%	b
UC-D (n=12)	51.6 ± 6.4	1.0 ± 0.5	37.5%	a, b

## Discussion

Recent research significantly reshapes our understanding of marginal integrity and mechanical strength in modern composite restorations. While traditional theories emphasized high polymerization shrinkage stress, 2024 finite element simulations suggest that these residual stresses are relatively low (<1 MPa) and are less likely to jeopardize restoration longevity than previously assumed. Clinical success is further dictated by the adhesive strategy employed [10]. A 2024 meta-analysis confirmed that universal adhesives (UAs) are highly versatile, yet their performance reaches its peak when applied via selective enamel etching (SEE) [11]. This study reinforces the critical role of material selection, demonstrating that UC-A (nano-hybrid) provides superior mechanical strength (125.4 MPa flexural strength) and marginal integrity (45.3 µm gap width) compared to UC-C. In terms of mechanical strength, the study in 2025 comparative analysis of high-viscosity bulk-fill composite resins (BFCRs) highlights a direct correlation between filler technology and functional performance [12]. Filtek One™ Bulk Fill demonstrated the highest flexural strength (142.5 MPa) and elastic modulus (10.5 GPa), attributed to its unique AUDMA and AFM monomers designed to reduce stress while maintaining high stiffness [13].

Microhardness is another important property, as it reflects surface resistance to indentation and wear. UC-A again outperformed other groups, while UC-C showed significantly lower hardness values. Similar trends were reported in a 2024 evaluation of universal composites, which concluded that microhardness is strongly dependent on filler morphology and degree of conversion [14,15]. Wear resistance testing confirmed these observations, with UC-A demonstrating the least volume loss and UC-C the highest. Wear resistance has a direct influence on clinical durability, especially in posterior teeth where occlusal forces are greatest. Different Studies emphasize that reduced wear is associated with higher filler content and improved salinization, supporting our findings for UC-A [16,17]. Marginal adaptation is essential for preventing microleakage, secondary caries, and restoration failure. In the present study, UC-A exhibited the smallest mean marginal gap width (45.3 µm) and the lowest leakage scores, while UC-C showed significantly larger gaps and more extensive dye penetration. This is consistent with evidence that polymerization shrinkage and stress distribution during curing vary across universal composites, influencing interfacial integrity [18,19].

The favorable sealing ability of UC-A may be attributed to lower polymerization shrinkage stress and balanced filler-resin ratios, whereas the poorer performance of UC-C may reflect higher shrinkage and weaker bonding at the interface. Similar findings were reported by other studies, which demonstrated that differences in photo initiator systems and resin chemistry significantly impact marginal adaptation outcomes [20]. From a clinical standpoint, the results indicate that while all tested materials are suitable for universal use, some offer superior durability and sealing ability under stress conditions. UC-A, in particular, combines higher flexural and hardness values with favorable marginal integrity, making it potentially more reliable for Class II restorations in high-stress regions. Conversely, composites such as UC-C may require careful consideration in stress-bearing situations due to lower strength and greater leakage tendency.

Study limitations

The complex oral environment cannot be fully replicated in this in-vitro investigation because important clinical factors such as pH and temperature changes, enzyme degradation, and masticatory fatigue were not replicated. Thermocycling was utilized to simulate aging; however, it is still a laboratory model that needs to be validated in a clinical setting. The study was further limited by a small sample size, evaluation of only four universal composites, and restriction to Class II cavity configurations. Future research should include a broader range of contemporary materials, extended aging protocols, advanced microstructural analyses, and long-term clinical trials to confirm mechanical performance and marginal integrity.

## Conclusions

The study concludes that UC-A (nano-hybrid) is the most effective universal composite among those tested, demonstrating superior mechanical strength (125.4 MPa flexural strength) and the most resilient marginal seal (45.3 µm gap width) following thermocycling. These results suggest that optimized filler technology and resin chemistry are vital for resisting masticatory forces and preventing microleakage.
